# Functional analysis of the *C*. *elegans cyld-1* gene reveals extensive similarity with its human homolog

**DOI:** 10.1371/journal.pone.0191864

**Published:** 2018-02-02

**Authors:** Paul Hadweh, Iro Chaitoglou, Maria Joao Gravato-Nobre, Petros Ligoxygakis, George Mosialos, Eudoxia Hatzivassiliou

**Affiliations:** 1 School of Biology, Aristotle University of Thessaloniki, Thessaloniki, Macedonia, Greece; 2 Department of Biochemistry, University of Oxford,South Parks Road, Oxford, United Kingdom; 3 Department of Medicine, School of Health Sciences, Aristotle University of Thessaloniki, Thessaloniki, Macedonia, Greece; Lund University, SWEDEN

## Abstract

The human cylindromatosis tumor suppressor (HsCyld) has attracted extensive attention due to its association with the development of multiple types of cancer. HsCyld encodes a deubiquitinating enzyme (HsCYLD) with a broad range of functions that include the regulation of several cell growth, differentiation and death pathways. HsCyld is an evolutionarily conserved gene. Homologs of HsCyld have been identified in simple model organisms such as *Drosophila melanogaster* and *Caenorhabditis elegans* (C. *elegans*) which offer extensive possibilities for functional analyses. In the present report we have investigated and compared the functional properties of HsCYLD and its *C*. *elegans* homolog (CeCYLD). As expected from the mammalian CYLD expression pattern, the CeCyld promoter is active in multiple tissues with certain gastrointestinal epithelia and neuronal cells showing the most prominent activity. CeCYLD is a functional deubiquitinating enzyme with similar specificity to HsCYLD towards K63- and M1-linked polyubiquiting chains. CeCYLD was capable of suppressing the TRAF2-mediated activation of NF-kappaB and AP1 similarly to HsCYLD. Finally, CeCYLD could suppress the induction of TNF-dependent gene expression in mammalian cells similarly to HsCYLD. Our results demonstrate extensively overlapping functions between the HsCYLD and CeCYLD, which establish the *C*. *elegans* protein as a valuable model for the elucidation of the complex activity of the human tumor suppressor protein.

## Introduction

Inactivating mutations in the human Cyld gene (HsCyld) predispose individuals to the development of skin tumors that include cylindromas, spiradenomas and trichoepitheliomas (reviewed in [1]). A tumor suppressing activity of HsCyld has been associated with several other types of human malignancies including multiple myeloma, melanoma, breast colon and hepatocellular carcinoma [2–6]. These findings have fueled an intense effort to understand the molecular mechanisms that underlie the homeostatic functions of HsCyld.

**Fig 1:**
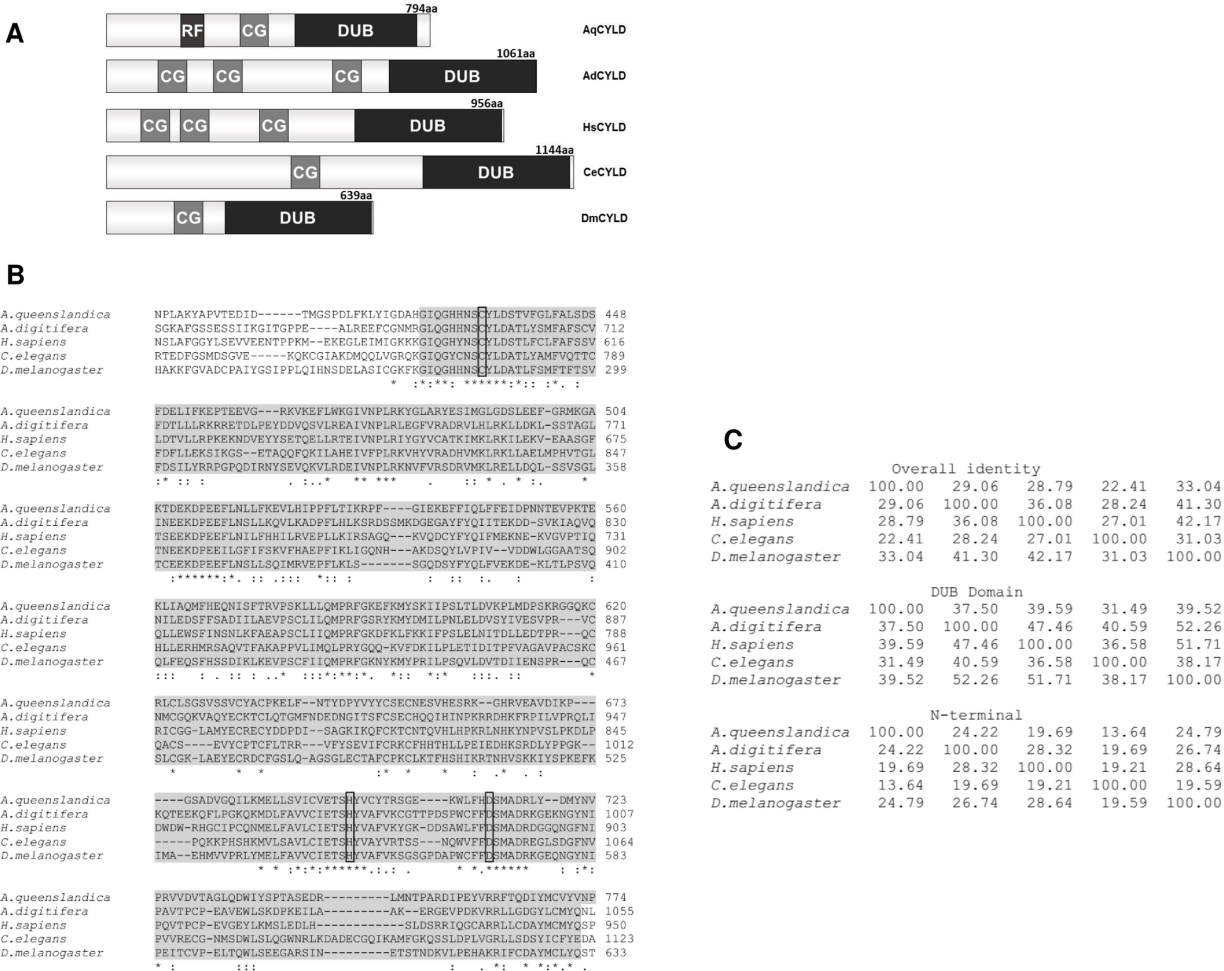
Structural organization and amino acid sequence comparisons of putative CYLD homologs from selected species that belong to five phyla. (A) Schematic representation of the structural organization of putative CYLD homologues from *A*.*queenslandica* (AqCYLD, Porifera), *A*.*digitifera* (AdCYLD, Cnidaria), *H*. *sapiens* (HsCYLD, Chordata), *D*. *melanogaster* (DmCYLD, Arthropoda) and *C*. *elegans* (CeCYLD, Nematoda). The relative position of a RING finger (RF), CAP-Gly (CG) and deubiquitinating (DUB) domains are shown. (B) Alignment of the deubiquitinating domain amino-acid sequences (shaded amino acids) of the putative CYLD homologues mentioned in A, using the Clustal Omega multiple sequence alignment software. The conserved cysteine, histidine and aspartate residues that form the catalytic triad are shown in boxes. The NCBI accession numbers of the protein sequences that were used for the alignment are the following: AqCYLD: XP_019849469.1, AdCYLD: XP_015748237.1, HsCYLD: CAB93533.1, DmCYLD: NP_609371.2 and CeCYLD: CAF31477.2. (C) Amino acid sequence identity values (%) from pairwise comparisons of full length (overall identity), deubiquitinating domain (DUB domain) and amino-terminal regions (N-terminal) of the putative CYLD homologues mentioned in A, as determined by the Clustal Omega multiple sequence alignment software.

HsCyld encodes a 956 amino acid polypeptide (HsCYLD) which has a carboxyl-terminal deubiquitinating domain and three amino-terminal CAP-Gly domains, two of which mediate the interaction of HsCYLD with microtubules (Fig 1A, [7]). Shorter variants of HsCYLD are predicted from multiple alternatively spliced mRNA species. HsCYLD preferentially hydrolyzes K63- and M1-linked polyubiquitin chains [8,9]. The deubiquitinating activity of HsCYLD has been associated with its ability to regulate several growth and survival pathways which include the NF-kappaB, JNK, p38, TGFbeta, Wnt and Notch pathways. The mechanism of HsCYLD-mediated inhibition of NF-kappaB and JNK activation by members of the TNF- and Toll/IL-1-receptor families has been studied more extensively than other signaling pathways. These studies have indicated that the inhibitory action of HsCYLD is mediated by its ability to hydrolyze polyubiquitin chains that may be free or conjugated onto specific proteins such as RIPK1, TAK1, TRAF-family members and Bcl3. The polyubiquitin chains that are targeted by HsCYLD mediate the assembly of multiprotein complexes that lead to proximity-induced activation of protein kinases and the propagation of signaling. Therefore, the hydrolysis of these polyubiquitin chains by HsCYLD disrupts the signaling process. By inhibiting NF-kappaB activation, HsCYLD can promote apoptosis which is inhibited by NF-kappaB. HsCYLD can promote also necroptosis, which is an alternative type of programmed cell death. The promotion of necroptosis by HsCYLD is based on its ability to deubiquitinate RIPK1 and facilitate its interaction with RIPK3 and the subsequent activation of MLKL. The ability of HsCYLD to facilitate various types of programmed cell death and inhibit growth and differentiation pathways is consistent with its broad tissues range of homeostatic functions.

Putative HsCyld homologues have been identified in multiple species including simple experimental model organisms such as *Drosophila melanogaster* (DmCyld) and *C*. *elegans* (cyld-1, called CeCyld hereafter) as well as evolutionarily more distant organisms belonging to phyla Cnidaria (*Acropora digitifera*, *Hydra Vulgaris* and *Nematostlella Vectensis*) and Porifera (*Amphimedon queenslandica*). The functional characterization of HsCyld homologues from model organisms will permit the use of genetically tractable organisms for the elucidation of the complex functional role of CYLD in animal pathophysiology. Fig 1 shows a schematic representation of HsCyld and its putative homologues from four representative species along with an amino acid alignment of their deubiquitinating domains and the results of pairwise comparisons of their amino acid sequences. These proteins have a similar domain organization with a highly conserved carboxyl-terminal deubiquitinating domain and amino terminal CAP-Gly domains. Previous work has demonstrated the ability of the Drosophila HsCYLD homologue to act as a deubiquitinating enzyme and regulate the IMD and JNK pathways in Drosophila, indicating the functional conservation between the human and fruit fly genes [10,11]. Furthermore, a recent evaluation of CeCyld-deficient *C*. *elegans* demonstrated an important role of CeCYLD in mediating p53-induced apoptosis similarly to the role of HsCYLD in preventing DNA-damage induced apoptosis [12]. However, a comparative biochemical and signaling characterization of CeCYLD and HsCYLD has not been performed. In the present report we have analyzed and compared the deubiquitinating and signaling properties of CeCYLD and HsCYLD and demonstrated an extensively overlapping functional pattern. Our findings support the use of *C*. *elegans* as a valid animal model for the exploration of the functional repertoire of HsCYLD.

## Materials and methods

### *C*. *elegans* strains

The following *C*. *elegans* strains were used:

CB6727: *unc-119(ed3) III; eEx657 [cyld-1p*::*DsRed2 + unc-119(+]*.

CB6753: *unc-119(ed3) III; eEx657 [cyld-1p*::*DsRed2 + unc-119(+]; rhIs4 (glr-1*::*GFP)*.

OH4120: *rhIs4 (glr-1*::*GFP) III; wrk-1(ok695) X*

The *cyld-1p*::*DsRed2* fusion was made by PCR amplification of a genomic DNA fragment that included 1.8Kb of the *cyld-1* upstream regulatory sequence followed by coding sequence terminating immediately after the second predicted exon (Fig 1). The PCR product was fused with DsRed2 and *unc-54* 3’ UTR from pMGN7[13]. The resulting fusion was then cloned into pJet1.2 to give pMGN27 and its sequence confirmed by Sanger sequencing. Transgenic strain CB6727 was generated by microinjection of the pMGN27 plasmid to *unc-119 (ed3)* mutants. Transgenic animals harboring the extrachromosomal array *eEx657* were crossed with transgenic worms expressing an integrated *glr-1*::*GFP* transgene *(rhIs4*). This strain was provided by the *Caenorhabditis* Genetic Center, which is funded by the National Institutes of Health—Office of Research Infrastructure Programs (P40 OD010440).

### Cell lines and transfection

HEK293T and HeLa cells were originally obtained from ATCC and cultured in Dulbecco’s modified Eagle medium (Gibco 41966029) supplemented with 10% FBS (Gibco 10270106), 1x Glutamax (Gibco 35050038), Penicillin (100 units/ml) and Streptomycin (100 μg/ml) (Invitrogen 15140–122). HEK293T cells were transfected using 2xBES (calcium phosphate transfection method) whereas HeLa cells were transfected using jetPRIME transfection reagent (Polyplus 114–07).

### Plasmids

Expression vectors for HsCYLD and TRAF2 were previously described [14,15]. The *C*. *elegans cyld-1* cDNA was prepared from total RNA obtained from mixed developmental stages of wild-type worms. In brief, total RNA was extracted using Trizol reagent (Thermo Fisher Scientific) and the first strand cDNA synthesis was performed using SuperScript VILO cDNA Synthesis Kit (Thermo Fisher Scientific), according to the manufacturer’s instructions. The 3.7 Kb *cyld-1* cDNA was subsequently synthesized via PCR with Phusion High-Fidelity DNA polymerase (New England Biolabs) using *cyld-1*-specific primers: Forw: 5’–atgcccggcgaatttgcaaagtttc-3’ and Rev: 5’-caactagttgaattacattcaccattctaag-3’. An amplicon, that corresponded to the *cyld-1 isoform a* (http://www.wormbase.org) was cloned into pJet1.2 to create pMGN32 and verified by sequencing. The latter was used as a template for PCR-based site directed mutagenesis. In order to introduce a mutation in the cysteine residue 774 of the CeCyld and replace it with serine, the BsiWI-ClaI fragment was mutagenized and used to replace the corresponding wild type fragment. The sequences of the mutagenic primers that were used are: For Mut 5’-TGTAACTCGTCTTATCTGGATGC-3’ and Rev Mut 5’-CCAGATAAGACGAGTTACAGTAACCC-3’, whereas the external primer pair used is For WT 5’- CAATGTGAATGGAAAGCAAGC-3’ and Rev WT 5’-TGACATGATCCGCTCGCACA-3’. The presence of the point mutation was verified by sequencing.

### Real-time PCR

HeLa cells were transfected with vectors expressing FLAG-tagged HsCYLD, CeCYLD or CeCYLDC774S. After 48h the cells were induced using 40ng/ml TNFα (Peprotech, 300-01A) for 1h. Total RNA was isolated from the cells using Nucleozol (Macherey-Nagel, 740404) and oligo-dT primed cDNA was prepared using the RevertAid Reverse Transcriptase (Thermo, EP0442) according to the manufacturer's instructions. For real-time PCR the IL8for (5′-CTGCGCCAACACAGAAATTA-3′) and IL8rev (5′-CTCTGCACCCAGTTTTCCTT-3′) primers were used for the amplification of the corresponding *IL8* mRNA fragment and the GAPDHfor (5′-AGCCACATCGCTCAGACAC-3′) and GAPDHrev (5′-GCCCAATACGACCAAATCC-3′) for the amplification of the corresponding *GAPDH* mRNA fragment. The expression levels of the transfected genes were verified by immunoblotting.

### Luciferase and β-galactosidase assays

HEK293T cells were cotransfected with a β-galactosidase expressing plasmid to test for transfection efficiency (pGKβ-gal [16]), and a plasmid containing the firefly luciferase gene under the control of three NF-kappaB binding sites (3xKBL [17]) or a plasmid containing the firefly luciferase gene under the control of seven AP1 binding sites (7xAP1, Stratagene) in the presence or absence of plasmids expressing FLAG-tagged TRAF2, HsCYLD, CeCYLD or CeCYLDC774S. The luciferase and β-galactosidase activities were determined by the Luciferase Assay System (Promega, E1501) and the Galacto-light Plus Reporter Gene Assay System (Applied biosystems, T1011) respectively. The cells were collected approximately 18 h after transfection and lysed in passive lysis buffer provided in the Tropix kit supplemented with 1mM DTT. The activities of the lysates were measured using a TD20/20 luminometer (Turner Designs). Relative luciferase activities were determined by dividing the values of luciferase activities by the corresponding values of β-galactosidase activities. The mean and the standard error were calculated using Microsoft Office Excel 2013 software.

### Immunoprecipitation and ubiquitin hydrolysis assay

HEK293T cells were cotransfected with vectors expressing FLAG-tagged HsCYLD, CeCYLD or CeCYLDC774S, using the calcium phosphate transfection method. The cells were harvested approximately 18 h post transfection and lysed in 1% NP40 lysis buffer (50mM Tris–HCL pH 7.4, 150mM NaCl, 1mM EDTA, 10% Glycerol, 1% NP-40, 1mM DTT, 1mM PMSF and 1× protease inhibitor cocktail (Roche, 1697498)). FLAG-tagged proteins were immunoprecipitated with the M2 anti-FLAG mouse monoclonal antibody (Sigma, F3165). The immunoprecipitated products were then divided equally and incubated *in vitro* with Poly-ubiquitin chains (Ub_3-7_, K63-linked) or Recombinant Human Tetra Ubiquitin/Ub4 WT Chains (M1 linked) (Boston Biochem, UC-320 and UC-710b respectively). The hydrolysis products were separated from the enzymes and boiled in 1xSDS loading buffer.

### Immunoblotting and silver staining

Transfected cells were lysed either as mentioned above or using RIPA buffer (50 mM Tris pH 7.5, 150 mM NaCl, 0.1% SDS, 0.5% sodium deoxycholate, 1% Triton-X 100, 1 mM EDTA, 1mM DTT, 1mM PMSF 1x protease inhibitor cocktail and for phosphorylated proteins 1x phosphatase inhibitor cocktail (Roche, 04906845001)). The following antibodies were used for immunoblotting: M5 anti-FLAG mouse monoclonal antibody (Sigma, F4042), β-Actin (C4) (Santa Cruz Biotechnology, sc-47778), anti-rabbit-HRP (Santa Cruz Biotechnology, sc-2004) and anti-mouse-HRP (Santa Cruz Biotechnology, sc-2005). The proteins were then treated with ECL-plus substrate (Thermo scientific, 32132) and detected using the imaging system Typhoon FLA 7000 (GE Healthcare). The ubiquitin hydrolysis products were loaded on 16% tricine gel and the products were detected by staining the gel with 0.15% silver nitrate.

## Results

### CeCYLD is a functional deubiqutinating enzyme with a broad pattern of expression

**Fig 2:**
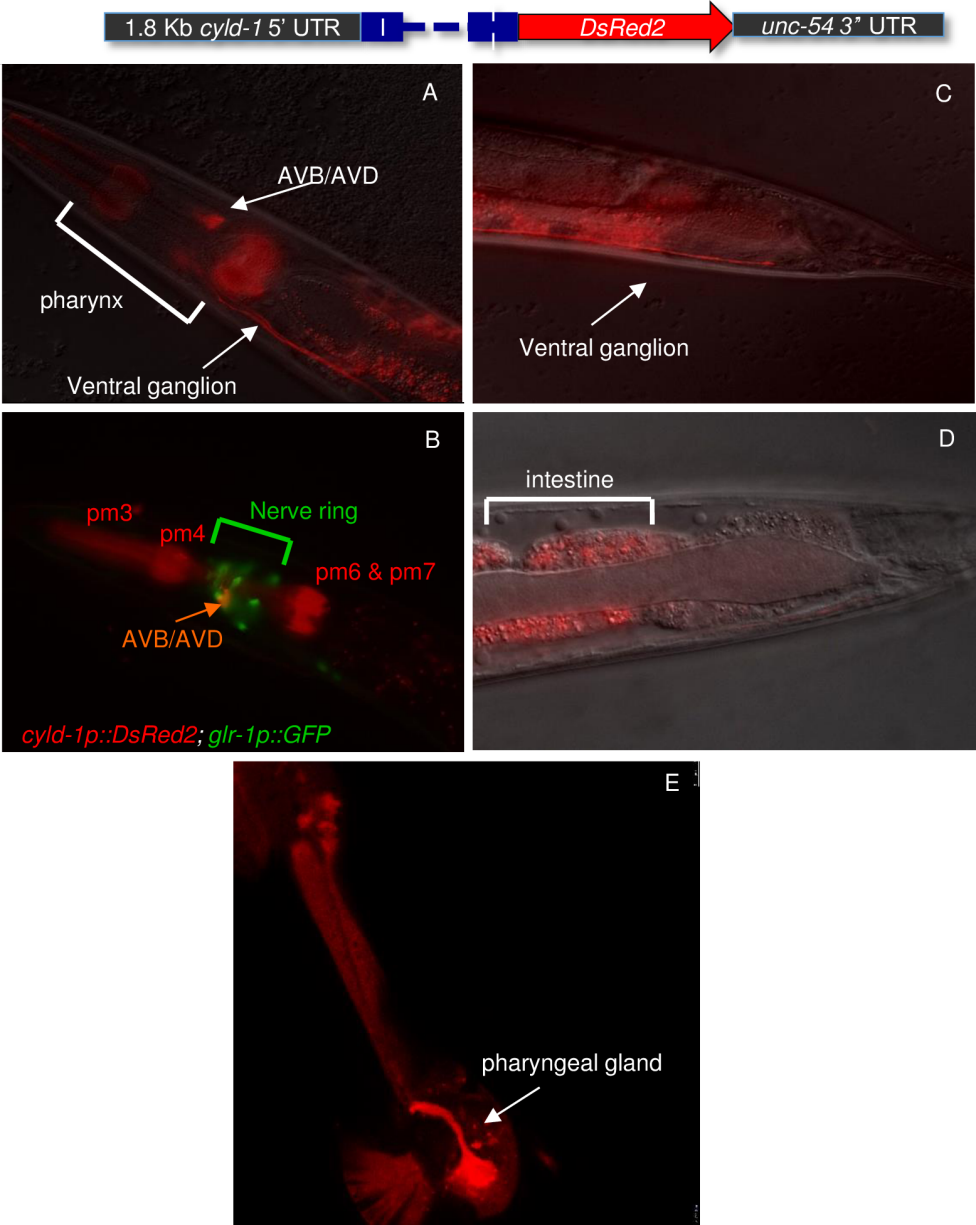
The *C*. *elegans cyld-1* expresses in the pharynx, in the nerve ring and in the intestine. Images of the head (A, B and E) and tail (C &D) region of transgenic worms that expressed the *cyld-1p*::*DsRed2* reporter. (A and B) Expression in the pharyngeal muscles pm3, pm4, pm6 & pm7. (B) The presumptive command interneurons AVB/AVD were identified using the *glr-1*::*GFP* reporter which expresses in command interneurons in the nerve ring. (C) AVB/AVD bundles run the entire length of the ventral nerve cord and terminate just before the rectum. (D) *cyld-1* expresses in the intestine. (E) Some animals show *cyld-1p*::*DsRed2* in the pharyngeal gland G2.

The expression pattern of CeCyld was evaluated to determine whether CeCyld has a broad or tissue specific function. Towards this goal transgenic worms that express DsRed under the control of the CeCyld promoter were generated (Fig 2). The analysis of the transgenic animal showed a rather broad pattern of reporter gene expression with prominent signals in the pharynx (Fig 2A, 2B and 2E), the presumptive AVB/AVD interneurons in the nerve ring (Fig 2B and 2C) and the intestine (Fig 2D). These findings are consistent with a functional role for CeCyld in multiple tissues similarly to the human gene.

**Fig 3:**
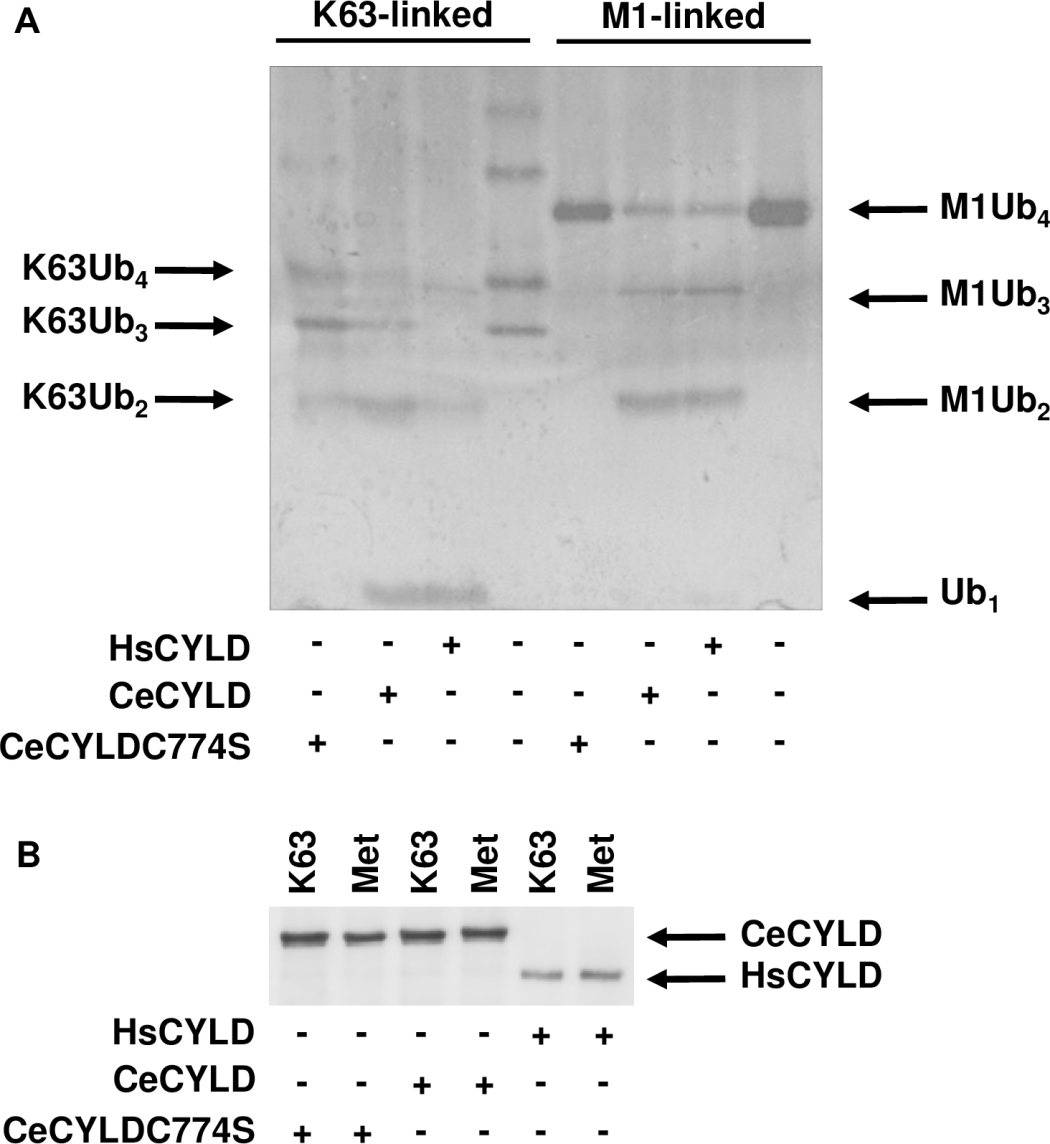
CeCYLD is a deubiquitinating enzyme with specificity for K63-linked and M1-linked polyubiquiting chains. HEK293T cells were transfected with vectors expressing the proteins indicated in the bottom of each figure. The cells were harvested approximately 18h post-transfection and lysed using NP-40 lysis buffer and the overexpressed proteins were immunoprecipitated as described above. The immunoprecipitated proteins were then incubated with M1-linked or K63-linked polyubiquitin chains. The immunoprecipitated proteins were then separated from the reaction products and analyzed separately by denaturing polyacrylamide gel electrophoresis. The reaction products were visualized by silver staining (A), whereas the immunoprecipitated proteins by immunoblotting using an anti-FLAG antibody (B). The position of monomeric ubiquitin (Ub_1_) or dimeric (Ub_2_), trimeric (Ub_3_) and tetrametic (Ub_4_) ubiquitin chains linked by K63 or M1 linkages are shown by arrows. The results of a representative experiment out of three are shown.

The extensive homology between the deubiquitinating domain of HsCYLD and the corresponding region of CeCYLD prompted an investigation into the ability of CeCYLD to act a deubiquitinating enzyme [18]. Towards this goal FLAG-tagged CeCYLD or HsCYLD were expressed in HEK293T cells and isolated by immunoprecipitation using an anti-FLAG antibody. The immunoprecipitated proteins were incubated with K63- or M1-linked polyubiquitin substrates and the products of the reaction were analyzed by electrophoresis. As shown in Fig 3 CeCYLD was capable of hydrolyzing both K63- and M1-linked chains with a similar efficiency to the human protein. The deubiquitinating activity of HsCYLD depends on several amino acid residues of the active site including cysteine 601 (C601). C601 of HsCYLD corresponds to C774 of CeCYLD (Fig 1B). In order to determine whether the catalytic activity of CeCYLD depends on C774 a mutated form of CeCYLD (CeCYLDC774S) containing a serine residue in place of C774 was generated and tested for its ability to hydrolyze K63- and M1-linked polyubiquitin chains. As shown in Fig 3 the deubiquitinating activity of CeCYLD towards both substrates was practically abolished by the replacement of C774 with serine. This result highlights an additional similarity between HsCYLD and CeCYLD and practically excludes the possibility of attributing the deubiquitinating activity of immunoprecipitated CeCYLD to an associated enzyme. Taken together these results demonstrate a comparable deubiquitinating activity and specificity between the human and *C*. *elegans* proteins, which strengthens further the evolutionary relationship of these proteins.

### CeCYLD inhibits the NF-kappaB and JNK activation pathways in human cells

**Fig 4:**
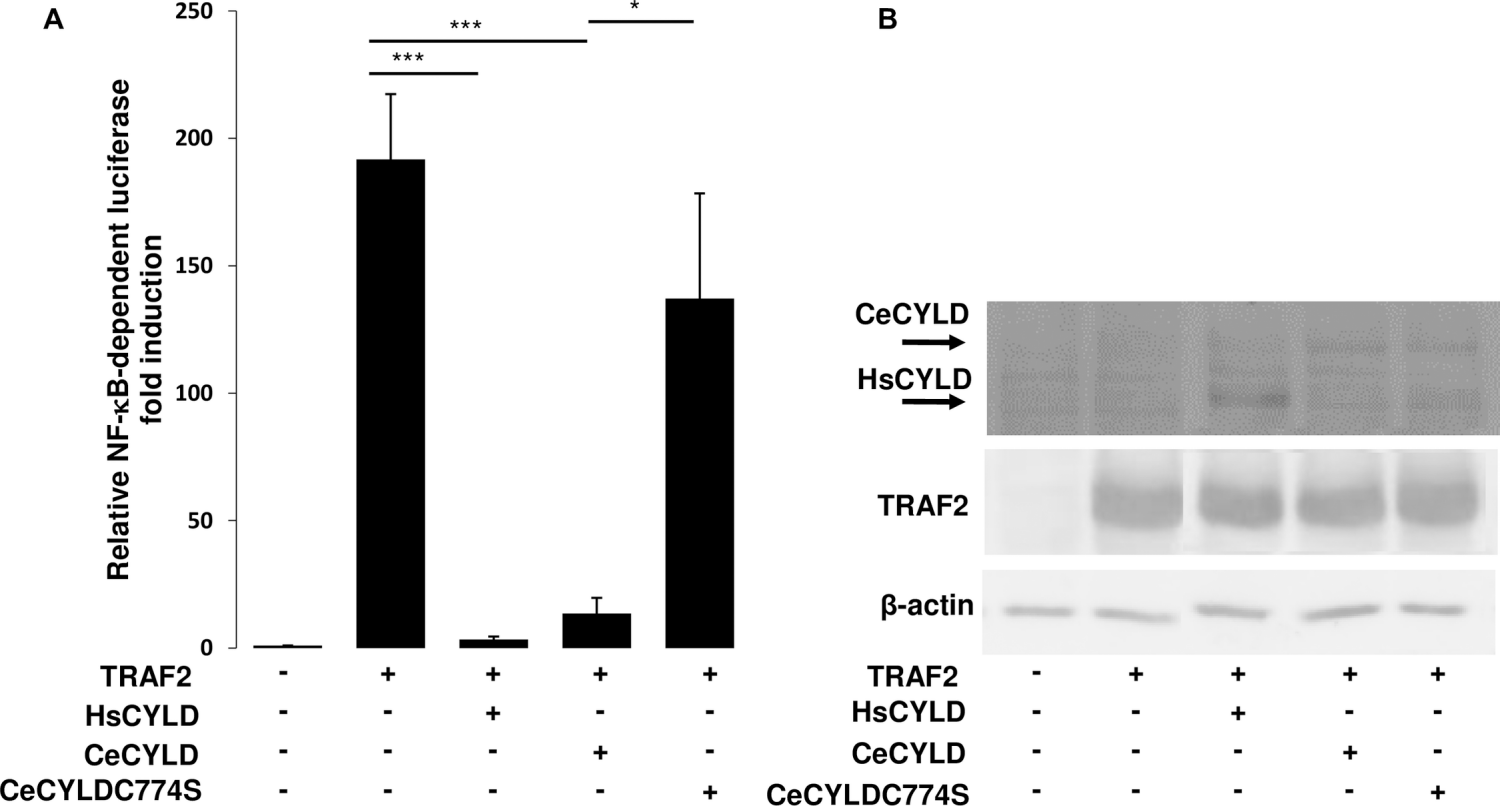
CeCYLD can inhibit the activation of NF-kappaB. HEK 293T cells (2x10^5^ cells per well in 12-well plates) were cotransfected with a NF-κB-dependent luciferase reporter plasmid (3XκBL), a β-galactosidase expression plasmid for transfection efficiency normalization (pGKβgal), and 0.4μg, 1.4μg and 0.4μg of plasmids expressing HsCYLD, CeCYLD and CeCYLDC774S respectively. After 18h, the cells were harvested and lysed in luciferase lysis buffer (Promega). The lysates were assayed for luciferase and β-galactosidase activities. (A) Values are shown as the mean +/- standard error of relative luciferase activity from six independent experiments. The values that were compared statistically are indicated by horizontal lines. (B) Representative immunoblot showing the expression levels of transfected proteins and β-actin. *p< 0.05, ***p<0.001.

**Fig 5:**
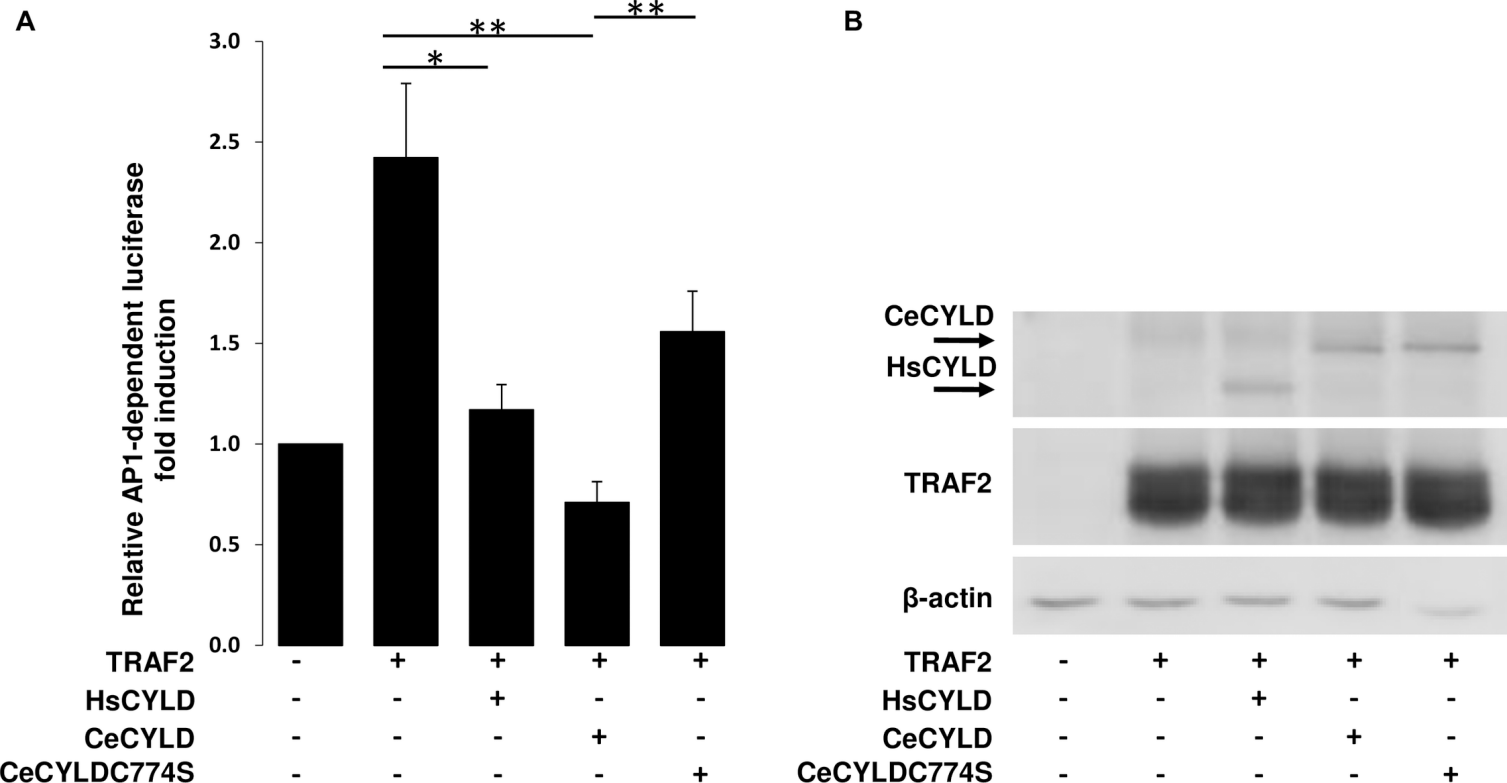
CeCYLD can inhibit the activation of AP1. HEK 293T cells (2x10^5^ cells per well in 12-well plates) were cotransfected with a AP1-dependent luciferase reporter plasmid (7xAP1), a β-galactosidase expression plasmid for transfection efficiency normalization (pGKβgal), and 0.1μg, 1μg and 0.3μg of plasmids expressing HsCYLD, CeCYLD and CeCYLDC774S respectively. After 18h, the cells were harvested and lysed in luciferase lysis buffer (Promega). The lysates were assayed for luciferase and β-galactosidase activities. (A) Values are shown as the mean +/- standard error of relative luciferase activity from five independent experiments. The values that were compared statistically are indicated by horizontal lines. (B) Representative immunoblot showing the expression levels of transfected proteins and β-actin. *p< 0.05, **p<0.01.

HsCYLD has a well-established role as an inhibitor of NF-kappaB and JNK activation pathways by members of the TNF and Toll/IL-1 receptor families. In order to explore further the functional relationship between CeCYLD and HsCYLD, the ability of CeCYLD to inhibit the NF-kappaB and JNK activation pathways was evaluated. The activation of these pathways was simulated in HEK293T cells by overexpression of TRAF2. The activity of NF-kappaB was monitored by a co-transfected NF-kappaB-dependent luciferase reporter plasmid (3xKBL). As shown in Fig 4 TRAF2 induced a significant activation of NF-kappaB-dependent luciferase activity, which was inhibited by the coexpression of HsCYLD as expected (Fig 4, S1 File). Coexpression of CeCYLD with TRAF2 also inhibited the activation of NF-kappaB similarly to HsCYLD despite the fact that CeCYLD was expressed at lower levels than HsCYLD. The inhibitory activity of CeCYLD on TRAF2-mediated NF-kappaB activation was dependent on its deubiquitinating activity since CeCYLDC774S was severely compromised in its ability to inhibit TRAF2-mediated activation of NF-kappaB. Immunoblotting demonstrated comparable expression of TRAF2 in these experiments. The JNK activation pathway was monitored by a cotransfected AP1-dependent luciferase reporter plasmid (7xAP1), since JNK activation induces the activity of AP1 transcription factor complex. As shown in Fig 5 TRAF2 induced a significant activation of AP1-dependent luciferase activity which was inhibited by the coexpression of HsCYLD as expected(Fig 5, S2 File). Coexpression of CeCYLD with TRAF2 also inhibited the activation of AP1 similarly to HsCYLD. The inhibitory activity of CeCYLD on TRAF2-mediated AP1 activation was dependent on its deubiquitinating activity since CeCYLDC774S was compromised in its ability to inhibit TRAF2-mediated activation of AP1. Immunoblotting demonstrated comparable expression of TRAF2 in these experiments. Collectively, these results expand the repertoire of similar functional properties between CeCYLD and HsCYLD.

### CeCYLD can inhibit TNF-dependent IL8 expression

**Fig 6:**
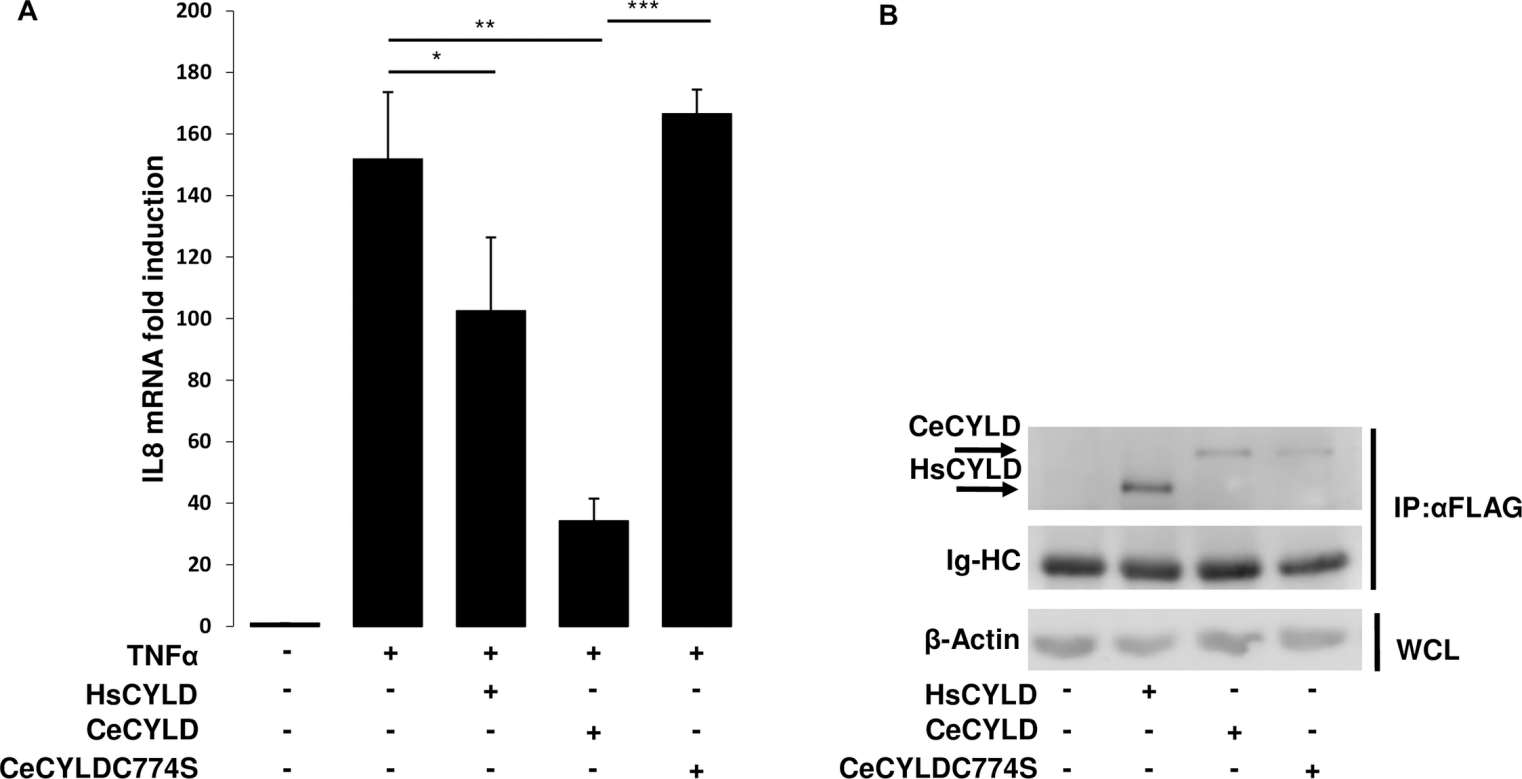
CeCyld inhibits TNF-induced IL8 expression. HeLa cells (10^5^ cells per well in 12-well plates) were transfected with 0.1μg, 1.4μg and 0.4μg of plasmids expressing HsCYLD, CeCYLD and CeCYLDC774S respectively. After 24h, each transfected cell population was split equally in two new wells and 24 h later, half of each transfected cell population was left untreated or treated with TNFα (40ng/ml, 1h). The cells were then harvested and from each cell population RNA and total protein was extracted. The overexpressed FLAG-tagged proteins were immuno-precipitated and the products of immunoprecipitation were used to assess the expression levels of each protein by immuoblotting. (A) Values are shown as the mean +/- standard error of relative IL8 expression induction from four independent experiments of RT-PCR. The values that were compared statistically are indicated by horizontal lines. (B) Immunoblot showing the expression levels of immuno-precipitated proteins with anti-FLAG antibody (IP:αFLAG) along with the immunoglobulin heavy chains (Ig-HC) and β-actin in the whole cell lysate (WCL). *p< 0.05, **p<0.01 ***p<0.001.

Our previous experiments indicated the ability of CeCYLD to interfere with the activation of the NF-kappaB and JNK signaling pathways similarly to HsCYLD. In order to determine whether CeCYLD could affect NF-kappaB-dependent endogenous gene expression, its ability to inhibit the induction of IL8 by TNF was evaluated in HeLa cells. For this purpose, HeLa cells were transfected with empty vector or vectors expressing HsCYLD or CeCYLD and stimulated with TNF. The relative expression of IL8 mRNA was determined by real time PCR. As shown in Fig 6, TNF stimulation caused a dramatic increase in the levels of IL8 mRNA (Fig 6, S3 File). As expected the exogenous expression of HsCYLD reduced the levels of TNF-induced IL8 mRNA. CeCYLD expression caused also a significant reduction to the levels of TNF-induced IL8 mRNA. On the other hand, CeCYLDC774S overexpression did not affect the induction levels of IL8 by TNFα (Fig 6). These results extend the functional similarity of HsCYLD and CeCYLD to include their common effect on NF-kappaB–driven endogenous gene expression.

## Discussion

The present report was undertaken to examine the extent of functional similarity between the human tumor suppressor protein HsCYLD and its *C*. *elegans* homologue CeCYLD. The two proteins have a relatively low overall amino acid sequence homology but a similar domain organization which includes amino terminal sequences with homology to CAP-Gly domains and a highly similar carboxyl terminal region with homology to deubiquitinating domains. Our experiments demonstrated that CeCYLD has a functional deubiquitinating domain with a similar substrate specificity to HsCYLD. In addition, CeCYLD was capable of inhibiting NF-kappaB and JNK pathway activation and TNF-induced gene expression in a manner that is similar to HsCYLD. These findings demonstrate an extensively overlapping range of functional properties between the worm and human proteins that support strongly an evolutionary relationship between these molecules.

KGB-1 is the JNK homologue in *C*. *elegans* [19]. It is primarily expressed in the nervous system where it controls coordinated movement. Furthermore *C*. *elegans* JNK plays important roles in stress responses to heavy metals, unfolded protein generation and bacterial pore forming toxins. Our findings on the expression pattern and activity of CeCYLD suggest that at least some of the functions associated with *C*. *elegans* JNK might be regulated by CeCYLD. Interestingly, a recent report demonstrated the importance of CeCYLD in the response of worms to DNA-damage induced apoptosis through the stabilization of the *C*. *elegans* homologue of p53 [12].

The activation of NF-kappaB by TNF- and Toll/IL-1-receptor families is another pathway that can be inhibited by HsCYLD and CeCYLD. Intriguingly, *C*. *elegans* has no known NF-kappaB protein homologues and therefore the biological significance of NF-kappaB activation inhibition by CeCYLD is unclear. However, it should be noted that the negative effects of HsCYLD on NF-kappaB activation are mediated by targeting signaling molecules functioning upstream from the NF-kappaB transcription factors (see introduction). Therefore, it is conceivable that CeCYLD may affect the activity of similar molecules that serve other functions in *C*. *elegans* and were later adopted to facilitate the activation of NF-kappaB in mammals. Possible targets of CeCYLD would include molecules that are implicated in the signaling of the *C*. *elegans* Toll-like receptor TOL-1 which is important for innate immune responses in the worm [20,21]. Therefore, it would be interesting to investigate whether CeCYLD might affect TOL-1 associated functions possibly in a ubiquitination-dependent manner. Likewise, HsCYLD homologues in Cnidaria and Porifera may regulate the signaling activity of Toll-like receptor, TRAF and NF-kappaB homologues that have been identified in these organisms [22].

Our experiments demonstrated a similar substrate specificity between CeCYLD and HsCYLD. These proteins can hydrolyze with similar efficiency K63-linked or M1-linked polyubiquitin chains. An examination of the amino acid sequence of the CeCYLD deubiquitinating domain is consistent with our findings and previously reported crystal structures. The catalytic triad of amino acid residues C-H-D which is conserved in all deubiquitinating enzymes of the USP class is conserved in CeCYLD and corresponds to amino acids C774, H1034 and D1050 (Fig 1, [8]). In accordance with this notion, our experiments demonstrated the functional significance of C774 for the deubiquitinating activity of CeCYLD. In addition, several structural features of HsCYLD and the zebrafish CYLD homologue, which are critical for the specific recognition of K63-linked and M1-linked polyubiquitin chains are conserved in CeCYLD. For example, the sequences of two short β-strands which are important for the specificity of zebrafish CYLD homologue towards K63-linked and M1-linked polyubiquitin chains are highly conserved in CeCYLD and correspond to amino acids 1053–1056 and 1062–1064 respectively [9]. The amino-terminal β-strand which spans amino acids 1053–1056 includes R1055 which was shown to be critical for the specific hydrolysis of K63-linked and M1-linked polyubiquitin chains by the zebrafish CYLD homologue. Furthermore, the shorter length of a loop between β-strands β6 and β7 in the zebrafish CYLD homologue is critical for the specificity of the enzyme towards K63-linked and M1-linked polyubiquitin chains [9]. The length and amino acid sequence of this loop is highly conserved in CeCYLD and spans amino acids 1030–1035.

Our study demonstrated for the first time the ability of CeCYLD to act as a deubiquitinating enzyme with similar activity and specificity to HsCYLD. In addition, a broadly overlapping spectrum of functions was demonstrated for the *C*. *elegans* and human proteins. These findings establish CeCyld as an orthologue of HsCyld and support the use of *C*. *elegans* as a valid model organism for the identification and elucidation of the functional properties of the human tumor suppressor gene by high throughput functional genomic analyses.

## Supporting information

S1 File Supplemental data for Fig 4A. The numerical values from six independent experiments (A-F) along with the corresponding average and standard error (std err) values and the t-test p-values that were used to generate the plot shown in Fig 4A are shown.
(PDF)

S2 File Supplemental data for Fig 5A. The numerical values from five independent experiments (A-E) along with the corresponding average and standard error (std err) values and the t-test p-values that were used to generate the plot shown in Fig 5A are shown.
(PDF)

S3 File Supplemental data for Fig 6A. The numerical values from four independent experiments (A-D) along with the corresponding average and standard error (std err) values and the t-test p-values that were used to generate the plot shown in Fig 6A are shown.
(PDF)
