# Supplementary material for: Functional analysis of the C. elegans cyld-1 gene reveals extensive similarity with its human homolog
Source: PLoS One. 2018 Feb 2;13(2):e0191864. doi: 10.1371/journal.pone.0191864 (PMC5796713; doi:10.1371/journal.pone.0191864)
Supplement: S1 File — The numerical values from six independent experiments (A-F) along with the corresponding average and standard error (std err) values and the t-test p-values that were used to generate the plot shown in Fig 4A are shown. (PDF) [file pone.0191864.s001.pdf]

	A	B	C	D	E	F	average	std err	*t* -test p-value
-	1,00	1,00	1,00	1,00	1,00	1,00	1,00	0,00	
TRAF2	155,37	257,60	128,56	125,72	259,07	224,66	191,83	25,58	
TRAF2 + HsCYLD	1,88	1,64	1,43	2,66	8,98	3,62	3,37	1,17	0,000645
TRAF2 + CeCYLD	4,30	1,42	8,33	3,77	25,66	38,15	13,60	6,08	0,000623
TRAF2 + CeCYLDC774S	69,80	66,79	156,56	22,68	277,19	229,81	137,14	41,30	0,019007
