# Supplementary material for: Functional analysis of the C. elegans cyld-1 gene reveals extensive similarity with its human homolog
Source: PLoS One. 2018 Feb 2;13(2):e0191864. doi: 10.1371/journal.pone.0191864 (PMC5796713; doi:10.1371/journal.pone.0191864)
Supplement: S2 File — The numerical values from five independent experiments (A-E) along with the corresponding average and standard error (std err) values and the t-test p-values that were used to generate the plot shown in Fig 5A are shown. (PDF) [file pone.0191864.s002.pdf]

	A	B	C	D	E	average	std err	*t* -test p-value
-	1	1	1	1	1	1,00		
TRAF2	1,43	3,17	1,64	3,07	2,8	2,42	0,37	
TRAF2 + HsCYLD	0,81	1,03	1,12	1,36	1,53	1,17	0,13	0,016
TRAF2 + CeCYLD	0,53	0,69	0,45	0,99	0,89	0,71	0,10	0,004
TRAF2 + CeCYLDC774S	1,21	1,86	1,07	1,5	2,15	1,56	0,20	0,005
