# Supplementary material for: Functional analysis of the C. elegans cyld-1 gene reveals extensive similarity with its human homolog
Source: PLoS One. 2018 Feb 2;13(2):e0191864. doi: 10.1371/journal.pone.0191864 (PMC5796713; doi:10.1371/journal.pone.0191864)
Supplement: S3 File — The numerical values from four independent experiments (A-D) along with the corresponding average and standard error (std err) values and the t-test p-values that were used to generate the plot shown in Fig 6A are shown. (PDF) [file pone.0191864.s003.pdf]

	A	B	C	D	average	std err	*t* -test p-value
-	1,0	1,0	1,0	1,0	1,0	0,00	
TNFα	198,3	104,5	127,2	177,6	151,9	21,74	
TNFα + HsCYLD	117,4	37,4	104,0	151,2	102,5	23,88	0,042267
TNFα + CeCYLD	28,2	18,0	38,6	52,1	34,2	7,28	0,00934
TNFα + CeCYLDC774S	153,0	152,8	178,5	181,9	166,5	7,90	3,19E-05
